# L-Theanine and Immunity: A Review

**DOI:** 10.3390/molecules28093846

**Published:** 2023-05-01

**Authors:** Shuna Chen, Jiaxin Kang, Huanqing Zhu, Kaixi Wang, Ziyi Han, Leyu Wang, Junsheng Liu, Yuanyuan Wu, Puming He, Youying Tu, Bo Li

**Affiliations:** Department of Tea Science, Zhejiang University, Hangzhou 310058, China; 22016068@zju.edu.cn (S.C.); 22116061@zju.edu.cn (J.K.); 22116067@zju.edu.cn (H.Z.); 22016169@zju.edu.cn (K.W.); 22216065@zju.edu.cn (Z.H.); 22216226@zju.edu.cn (L.W.); 0022326@zju.edu.cn (J.L.); yywu@zju.edu.cn (Y.W.); pmhe@zju.edu.cn (P.H.); youytu@zju.edu.cn (Y.T.)

**Keywords:** L-theanine, immunoregulation, mechanism, application

## Abstract

L-theanine (N-ethyl-γ-glutamine) is the main amino acid in tea leaves. It not only contributes to tea flavor but also possesses several health benefits. Compared with its sedative and calming activities, the immunomodulatory effects of L-theanine have received less attention. Clinical and epidemiological studies have shown that L-theanine reduces immunosuppression caused by strenuous exercise and prevents colds and influenza by improving immunity. Numerous cell and animal studies have proven that theanine plays an immunoregulatory role in inflammation, nerve damage, the intestinal tract, and tumors by regulating γδT lymphocyte function, glutathione (GSH) synthesis, and the secretion of cytokines and neurotransmitters. In addition, theanine can be used as an immunomodulator in animal production. This article reviews the research progress of L-theanine on immunoregulation and related mechanisms, as well as its application in poultry and animal husbandry. It is hoped that this work will be beneficial to future related research.

## 1. Introduction

Tea has been a very popular beverage throughout the world for centuries and contains 26 amino acids (6 non-protein amino acids), accounting for 1–5% of the dry weight of tea. L-theanine, systematically named N-ethyl-γ-L-glutamine, is a non-protein, water-soluble amino acid that accounts for more than 50% of the total free amino acids in tea leaves. The biosynthesis of L-theanine is based on the synthesis of glutamic acid and ethylamine by theanine synthetase in the root of the tea tree, and then L-theanine is transported through the bast and accumulated in the leaves. As a characteristic amino acid in tea, L-theanine contributes a lot to the fresh and caramel flavor of tea and relieves the bitter taste of caffeine [1,2]. The main physicochemical properties of L-theanine are shown in Table 1. After oral administration, L-theanine is rapidly absorbed in the intestine, mainly through the sodium-coupled co-transporter in the intestinal brush margin mucosa. Then L-theanine acts on various tissues and organs through blood circulation and crosses the blood-brain barrier to act on the brain, thus regulating body functions. Finally, L-theanine is excreted from urine or metabolized by enzymatic hydrolysis in the kidneys to glutamic acid and ethylamine, which are excreted in urine [1]. A pharmacokinetic study in humans showed that the lag time of L-theanine intake was about 10 min, and the half-lives of absorption and elimination were about 15 min and 65 min, respectively. After about 50 min, L-theanine reached its maximum blood concentration [3]. In rats, the concentration of L-theanine reached a peak in serum and liver 1 h after intragastric administration (4 g/kg) and then gradually decreased. It reached the highest level in rat brain tissue 5 h after administration and disappeared completely in the brain 24 h after administration [4].

The safety of L-theanine has been evaluated in several toxicity studies. The acute oral maximum tolerance dose (MTD) of L-theanine was greater than 20.0 g/kg in both male and female mice. According to the standard for the classification of acute toxicology, it was regarded as non-toxic [5]. In a subchronic toxicity study, L-theanine was administered to female and male rats at 0, 1.5, 3, or 4 g/kg for 13 weeks. No adverse effects on organ weight or histopathology were observed [6]. In chronic toxicity tests, B6C3F1 mice did not show any chronic toxicity or tumorigenicity at the oral maximum tolerance dose of L-theanine for 78 weeks [7]. Data from the above animal studies suggest that L-theanine is absorbed and eliminated in humans, and high doses of L-theanine are relatively safe.

Naturally occurring theanine in tea is L-configuration, while synthetic theanines are a mixture of D- and L-configuration. The biological activity of L-theanine in vivo is much higher than that of D-theanine [8]. As one of the major active ingredients in tea and a new food additive, L-theanine has been found to possess many health benefits, such as anti-oxidation [9], anti-inflammation [10], neuroprotection [11], anti-anxiety [12], anti-cancer [13], anti-obesity [14], metabolic regulation [15], cardiovascular protection [16], liver and kidney protection [17], and immune regulation [18]. Compared with other biological activities, the immunomodulatory function of L-theanine has received less attention [19]. This work reviews the in vitro, animal, clinical, and epidemiological studies of L-theanine in improving immunity, discusses the relevant mechanisms, and summarizes its application in poultry and animal husbandry. It is hoped that this review will provide useful information for future research on L-theanine immunity. 

## 2. Clinical and Epidemiological Studies on Immune Regulation by L-Theanine

### 2.1. Alleviation of Immunosuppression Caused by Strenuous Exercise

Since the end of the last century, epidemiological studies have shown a link between L-theanine and the alleviation of immune suppression induced by strenuous exercise. Athletes fail to recover adequately after a period of intense, high-load exercise, which is called overtraining syndrome. This is because chronic fatigue and decreased physical performance weaken the immune system, resulting in increased neutrophils and decreased lymphocytes in the blood, which in turn leads to inflammation, immunosuppression, and reduced resistance to disease (diarrhea, fever, pharyngitis, and colds). Oral supplementation of L-theanine and cystine can alleviate the fluctuation of blood immunocompetent cells caused by high-intensity endurance exercise, inhibit the excessive inflammatory response, prevent various infectious diseases, reduce related muscle damage, and alleviate immunosuppression [20].

Resistance exercise training increases muscle strength but also has negative effects on the immune system, such as decreased lymphocyte proliferation, reduced T-helper lymphocyte counts, and decreased natural killer (NK) cell activity. In the immune system, NK cell activity is considered one of the important indicators for monitoring immunity and plays an important role in innate immunity. Training experiments have shown that L-theanine and cystine supplementation can increase glutathione (GSH) levels, restore NK cell activity reduced by high-intensity and high-frequency resistance exercise, and enhance immune responses [21]. L-theanine supplementation also contributes to the interleukin (IL)-10 reduction after exercise, which has a beneficial effect on regulating the helper T lymphocyte (Th)1/Th2 balance that is disrupted after strenuous exercise [22].

### 2.2. Prevention of Colds and Flu

L-theanine is able to protect against the common cold and flu. Randomized, double-blind, placebo-controlled trials have shown that the incidence of colds in subjects with cystine and L-theanine supplementation was significantly lower than that in the placebo group. L-theanine enhanced γδT cell function, promoted interferon (IFN)-γ secretion, and reduced the incidence of cold and fever symptoms [23,24]. In health care workers, supplementation with L-theanine or catechin for 5 months significantly protects against influenza and is well tolerated [25]. Older people have a weaker immune response to influenza vaccines and are at higher risk of illness and death from influenza viruses. Studies have reported that supplementation of L-theanine and L-cystine before vaccination can enhance the immune response to the influenza vaccine, improve the vaccination effect, and enhance the immune protection ability of the elderly with low hemoglobin levels in the serum [26].

### 2.3. Promotion of Postoperative Recovery

Perioperative regulation of hyperinflammatory and immunosuppressive states is important to achieve a stable postoperative process and early recovery and to reduce the recurrence rate of malignant tumors. Local inflammation caused by surgery becomes systemic within 4–8 h and induces systemic inflammatory response syndrome (SIRS), leading to increased levels of proinflammatory cytokines and neutrophil infiltration and causing tissue and organ damage. GSH is a powerful antioxidant in the body and is important for the immune system. For gastric cancer patients undergoing distal gastrectomy, the GSH levels in the blood and skeletal muscle decreased after surgery. Oral administration of L-theanine and cystine in the perioperative period increased GSH level, inhibited the increase in resting energy expenditure (REE), promoted the rapid recovery of body temperature, IL-6 level, C-reactive protein (CRP) level, neutrophil count, and total lymphocyte percentage, reduced inflammation after gastrectomy, and promoted postoperative recovery [27]. For patients with colorectal cancer and gastrointestinal cancer receiving postoperative drug chemotherapy, oral administration of L-theanine and cysteine is safe and able to reduce serious adverse events caused by chemotherapy, alleviate the incidence of diarrhea or hand-foot syndrome (HFS), reduce peripheral neuropathy induced by chemotherapy drugs, and improve the sufferer’s quality of life [28,29,30].

## 3. Studies on Immunomodulatory Effects of L-Theanine In Vitro and in Animal Models

### 3.1. Reduction of Inflammation

During the infection phase, the innate and adaptive immune systems engage in an inflammatory response, through which most infections are cleared. Inflammation is also triggered after surgery in response to protecting the body from infection or trauma. However, persistent and excessive inflammatory responses, such as SIRS, trigger decreased immune function or multi-organ failure. In mice, preoperative oral administration of L-theanine and cystine inhibited the surgically induced decline in intestinal GSH levels, inhibited IL-6 production and excessive inflammatory responses, and promoted spontaneous motor activity, postoperative eating, and early postoperative recovery [31].

When the degree and duration of intestinal hypoperfusion are severe and long, intestinal reperfusion may aggravate organ damage by excessive activation of polymorphonuclear neutrophils, oxidative stress, and pro-inflammatory mediators and trigger inflammatory responses. A variety of therapeutic approaches have been proposed to mitigate intestinal ischemia-reperfusion injury. Preoperative oral administration of L-theanine and cystine regulates the oxidation-reduction cycle of GSH, decreases the inflammatory cytokines, alleviates the infiltration of white blood cells, and reduces the damage of inflammatory injury to the body tissues, thus improving the survival rate after intestinal ischemia-reperfusion in a dose-dependent manner [32].

In a mouse model of psoriasis-like skin inflammation induced by imiquimod (IMQ), L-theanine significantly reduced the inflammatory response and down-regulated the expression of keratin 17, IL-23, and C-X-C motif chemokine ligand 13 (CXCL13). Furthermore, L-theanine inhibited the production of IL-23 in dendritic cells (DCs) after IMQ treatment and decreased the levels of chemokines in keratinocytes treated with IL-17A by downregulating IL-17RA expression [33]. L-theanine ameliorated 12-O-tetradecanoylphorbol-13-acetate (TPA)-induced erythema, increased vascular permeability, epidermal and dermal hyperplasia, and neutrophil infiltration via decreasing platelet endothelial cell adhesion molecule (PECAM)-1 expression and the production of pro-inflammatory cytokines including IL-1β, tumor necrosis factor (TNF)-α and cyclooxygenase (COX)-2 [34].

Liver cirrhosis is closely associated with oxidative stress, chronic inflammation, and increased transforming growth factor (TGF)-β expression. L-theanine protected the liver from fibrosis and inflammatory damage induced by carbon tetrachloride (CCl4) in an experimental cirrhotic rat model. L-theanine reduced the levels of TGF-β and connective tissue growth factor (CTGF) and induced the activation of matrix metallopeptidase (MMP)-13, thus inhibiting liver fibrosis. In addition, it is able to inhibit nuclear factor (NF)-κB (a well-known target of liver injury), thereby reducing proinflammatory cytokines (IL-1β and IL-6) and increasing anti-inflammatory cytokines (IL-10), indicating it has anti-inflammatory and anti-necrosis activities [35].

Asthma is a chronic inflammatory and allergic disease of the airway. Exposure to allergens can induce various inflammatory responses. L-theanine has an inhibitory effect on ovalbumin (OVA)-induced airway inflammation in allergic asthma. It can significantly inhibit the secretion of TNF-α, IFN-γ, IL-4, IL-5, IL-13, and monocyte chemoattractant protein (MCP)-1 induced by OVA, inhibit the oxidative stress response, and suppress the activation of the NF-κB pathway [36].

Osteoarthritis (OA) is often characterized by low-grade inflammation. In IL-1β-stimulated rat chondrocytes, L-theanine reduced the release of catabolic enzymes and inflammatory mediators from chondrocytes, inhibited the up-regulation of MMP-3 and MMP-13, and protected against extracellular matrix degradation. In addition, it can reduce the release of inflammatory cytokines, including COX-2, prostaglandin E2 (PGE2), inducible nitric oxide synthase (iNOS), and nitric oxide (NO), by inhibiting the NF-B pathway. In the rat model of anterior cruciate ligament transection (ACLT), L-theanine improved knee histopathology, reduced extracellular matrix (ECM) degradation, and decreased the levels of proinflammatory mediators in a dose-dependent manner. L-theanine at 200 mg/kg showed a similar treatment effect compared with the same dose of celecoxib [37].

### 3.2. Alleviation of Nerve Injury

Nerve injury is generally accompanied by neuroinflammation, oxidative stress, local ischemic necrosis, neuronal apoptosis, etc., and is closely associated with the immune system of the organism. Spinal cord injury (SCI) is a devastating condition that causes nerve damage and impairs mobility. L-theanine treatment significantly reduced the levels of NO and malondialdehyde (MDA), increased the antioxidant ability, and inhibited the levels of neuroinflammatory and apoptotic markers, thus protecting the body from nerve damage caused by SCI and improving the recovery of behavioral and motor functions [38]. In a rat model of brain nerve injury induced by aroclor 1254, L-theanine increased the antioxidant capacity in the brain, reduced the levels of lipid hydroperoxide (LPO) and NO, enhanced the activities of creatine kinase (CK), acetylcholinesterase (AchE), and ATPases, restored the normal structure of the brain region, and downregulated the expression of inflammatory cytokines, thus protecting the brain nerve against the oxidative damage [39].

Cadmium (Cd) has long been known to cause neurodegenerative diseases. In Cd-induced neurotoxicity mice, oral administration of L-theanine can reduce the Cd levels in the brain and plasma, inhibit the apoptosis of neurons in the cerebral cortex and hippocampus, reduce the levels of MDA and reactive oxygen species (ROS) in brain tissue, increase the GSH level and the activities of superoxide dismutase (SOD), catalase (CAT), and glutathione peroxidase (GSH-Px), and alleviate oxidative damage. At the same time, L-theanine attenuated tau protein hyperphosphorylation by inhibiting the activation of glycogen synthase kinase (GSK)-3β and Akt/mTOR signaling pathways and protected mice from Cd-induced neurotoxicity [40]. In a rat model of Alzheimer’s disease induced by amyloid β, luteolin combined with L-theanine can inhibit TNF-α, reduce neuroinflammation, and improve memory function [41]. Similarly, krill oil combined with nobeletin and L-theanine ameliorated memory and cognitive deficiency in senescence-accelerated prone mouse/8 (SAMP8), showing synergistic effects on inhibition of Aβ aggregation, neurofibrillary tangles, apoptosis, and neuroinflammation [42]. 

### 3.3. Improvement of Intestinal Immunity

The gut is the largest immune organ in the human body and plays a central role in regulating immune homeostasis. Although chemotherapy is an effective cancer treatment, many types of anticancer drugs not only inhibit the proliferation of cancer cells but also attack normal intestinal mucosal cells, leading to adverse events such as intestinal mucositis and the destruction of intestinal immunity. The combination of L-theanine and cystine (5:2, *w/w*) can reduce the shortening of intestinal villi and the crypt destruction induced by 5-FU, prevent the reduction of GSH level, inhibit ROS production and oxidative stress, and thus inhibit intestinal mucositis and diarrhea [43]. Radiotherapy also causes severe intestinal damage and bone marrow suppression. Small intestinal crypt cells are particularly sensitive to radiation and prone to apoptosis. Pretreatment with L-theanine and cysteine can prevent the shortening of villus length, increase the depth of the crypt, and reduce the number of apoptotic cells in the jejunal crypt after irradiation [44].

Production of antigen-specific IgE antibodies and disruption of the gut microbiota are the main features of OVA-induced immediate hypersensitivity. L-theanine treatment alleviates allergic symptoms, decreases the levels of IgE, HIS, and mast cell protease-1 (mMCPT-1) in serum, reduces jejunal inflammation and MC degranulation, regulates the differentiation of jejunal CD^4+^ T cells to Th1 and Th17 cells, inhibits the differentiation of Th2 and Treg cells and their cytokines, and improves gut-specific immunity. In addition, L-theanine increases intestinal microbial diversity and alleviates OVA-A pathological symptoms by regulating the intestinal microbiota and its short-chain fatty acid metabolites [45].

A healthy gut is important for poultry growth. L-theanine is able to promote the diversity of intestinal microorganisms, increase the relative abundance of beneficial intestinal microflora, and inhibit the harmful bacteria in the digestive tract, thereby benefiting the balance of intestinal microecology and promoting intestinal immunity. In addition, L-theanine directly reduced the mRNA expression of Toll-like receptors (TLRs) (TLR-2, TLR-4) and inflammatory cytokines (TNF-α, IFN-γ, and IL-2), enhanced the proliferation of T lymphocytes and regulated the intestinal mucosal immune response and inflammation. L-theanine supplementation improved tight junction (TJ) barrier integrity by enhancing the expression of ZO-1, occluding, and claudin-3 in the intestine [46].

### 3.4. Regulation of Tumor Immunity

Lack of immune surveillance and immune escape are important reasons for the generation and development of tumor cells. Dendritic cells (DC) are powerful antigen-presenting cells in the body and play an important role in tumor immunosuppression. Tumor cells can escape the host immune surveillance system, allowing tumor growth and metastasis. A tumor has an inhibitory effect on DC, which can cause immune tolerance and loss of the specific killing target of T cells. Studies have shown that the co-culture of DC cells with bombesin, a cytokine secreted by tumor cells, significantly decreases the number of DC and causes DC dysfunction by reducing the expression of HLA-DR, CD86, CD83, CD80, and CD40 in DC. L-theanine can antagonize the inhibition of DC by bombesin, improve the ability of DC to stimulate the proliferation of T lymphocytes, enhance the antigen presentation activity of DC, activate the function of the suppressed immune system, and then inhibit the growth and metastasis of lung cancer cells. Further studies showed that L-theanine inhibited the transcription and translation of COX-2 and reduced endogenous IL-10 synthesis. Meanwhile, it promoted DC maturation, thereby increasing the secretion of IL-12 and regulating the balance between IL-10 and IL-12. L-theanine can enhance the ability of immune surveillance and inhibit the activation of protumor substances by enhancing the ability of phagocytosis and presentation of antigen substances from DC, thus achieving the anti-lung adenocarcinoma effect [47].

For highly metastatic lung cancer cells, human cervical cancer cells, and hepatocellular carcinoma cells, L-theanine and its derivatives effectively inhibited cell growth and migration in vitro and in vivo by targeting the EGFR/VEGFR-Akt/NF-κB pathway. They decreased the expression of CD44, reduced the phosphorylation or expression of EGFR, Met, Akt, and NF-κB, completely inhibited EGFR/MET-Akt/NF-κB signaling activated by HGF and EGF, and showed a higher inhibition effect on cancer cells when combined with anticancer drugs cytarabine, pyirubicin, vincristine, and methotrexate [48,49,50,51].

L-theanine has a potent cytotoxic effect on human HepG2 hepatoblastoma and HeLa adenocarcinoma cell lines. Further experiments showed that L-theanine caused the loss of membrane potential and the release of apoptosis-inducing factors, endonuclease G and cytochrome c, through the mitochondrial pathway and caused tumor cell apoptosis by activating caspase-9 and caspase-3 [52,53,54,55,56,57]. In the DMH-induced colon cancer model, L-theanine can prevent tumorigenesis by down-regulating the expression of Akt and mTOR, inhibiting the JAK2/STAT3 pathway, and increasing the expression of Smad2, which is a tumor suppressor [58]. For prostate cancer, L-theanine can inhibit invasion and migration and increase intercellular adhesion of prostate cancer cells in vitro and in vivo. L-theanine down-regulated the expressions of MMP9, N-cadherin, vimentin, and Snail, up-regulated E-cadherin, and significantly inhibited the ERK/NF-κB signaling pathway and the binding activity of p65 to the promoter regions of MMP9 and Snail [59].

## 4. Immunomodulatory Mechanisms of L-Theanine

### 4.1. Activation of γδT Lymphocyte Function

Numerous studies have shown that L-theanine can enhance the function of γδT cells in the immune system. γδT cells are a class of T lymphocytes that perform innate immune functions, mainly expressing CD^3+^CD^4−^CD^8−^. γδT cells are the main subsets that regulate and initiate anti-infection immune responses and secrete a variety of cytokines such as IL-2, IL-3, IL-6, IFN-γ, TNF-α, etc. They regulate specific immune responses and are the first line of defense against microorganisms and tumors. In vitro experiments showed that alkylamine antigen caused γδT cells to proliferate and secrete IL-12-dependent IFN-γ cytokines. In vivo experiments showed that alkyl amines in tea activated the human innate immune response. L-theanine is a nonprotein bioactive substance containing alkyl amines that can be quickly and directly recognized by γδT cells. Due to the non-MHC restriction of γδT cells to antigens and the absence of antigen processing and presentation recognition, a large number of memory Vγ2Vδ2 T cells can stimulate immune responses by recognizing alkylamine antigens. L-theanine is decomposed into ethylamine in the human liver, which can be contacted by γδT cells in the peripheral blood. It can regulate the function of γδT cells, enhance the human innate immune response, and reduce the occurrence of influenza and other diseases [59,60]. Supplementation of capsules containing L-theanine and catechin reduced the incidence of cold and flu symptoms by enhancing γδT cell function [61]. In the elderly with poor nutrition, antibody production is significantly inhibited after vaccination. L-theanine can be broken down into glutamic acid and ethylamine. Glutamate and L-cystine convert to glutathione, which stimulates γδ T cells to release IL-2 and restores impaired immune function [26].

L-theanine may activate γδ cells by inhibiting mevalonate metabolism. The mevalonate pathway (MEP) is a metabolic pathway that uses acetyl-coA as a raw material to synthesize isoprene pyrophosphate (IPP) and dimethylallyl pyrophosphate (DMAPP), which play an important role in regulating cell growth, differentiation, and proliferation. L-theanine and other alkyl amines are absorbed by peripheral blood mononuclear cells (PMBC) and specifically inhibit the activity of fartnyl pyrophosphate (FPP) synthetase in the MEP, leading to the accumulation of IPP located upstream of the signaling pathway. IPP is an antigen receptor agonist of γδT cells and can be recognized as the first antigen by γδT cells. Excessive accumulation of IPP activates γδT cells, thereby secreting IFN-y cytokines, killing infected cells, presenting antigen to αβT cells, and initiating an immune response. It also forms immune memory and increases the recognition and killing of non-specific antigens (such as tumors, viruses, pathogens, etc.). The intake of alkylamines through diet (L-theanine) and medicines (such as bisphosphonates) inhibits the MEP and increases the accumulation of IPP so that Vγ2Vδ2T cells are in the primed and ready state, which can rapidly respond and proliferate in the face of pathogens and expand up to 50 times in the peripheral blood [53]. It has also been proven that L-theanine regulates the secretion of cytokines in spleen lymphocytes by activating the expression of Rap1A and 3-hydroxy-3-methylglutaryl-CoA reductase (HMGCR) proteins in the MEP and is a promising immunostimulant [62].

### 4.2. Promotion of GSH Synthesis and Antioxidant Capacity

L-theanine is metabolized to glutamate in the liver, and cystine is a dipeptide of cysteine. Glutamate and cysteine are considered beneficial for the immune system because they are involved in the synthesis of reduced glutathione (GSH). In vitro, administration of glutamate and cystine can enhance the GSH concentration in immune cells. In vivo, supplementation of L-theanine and cystine increases the GSH concentration in the liver, enhances antioxidant capacity, improves the Th2-mediated immune response, promotes the production of antigen-specific IgG, and heightens the immune function of the body [63,64].

The immune function of the human body weakens with age, and the intracellular GSH level also decreases. Studies have shown that L-cystine and L-theanine increase the production of antigen-specific IgM and IgG in aged mice and significantly enhance the mRNA expression of glutamyl cysteine synthase (the rate-limiting enzyme for GSH synthesis) in the spleen [65]. In aging rats induced by D-galactose, L-theanine improves oxidative stress and inflammatory response, reduces the level of advanced glycation end products (AGEs), maintains homeostasis, and delays liver aging [66].

Excessive alcohol intake causes alcoholic liver damage (ALD), which is related to the formation of lipid free radicals and lipid peroxidation. Ingestion of L-theanine enhances the antioxidant capacity of hepatocytes, inhibits the production of alanine transaminase (ALT), aspartate aminotransferase (AST), triglyceride (TG), ROS, and MDA, increases the activities of SOD, CAT, and GR, and increases the GSH level. In addition, L-theanine inhibits caspase-3 reduction and poly (ADP-ribose) polymerase (PARP) cleavage, suppresses the collapse of mitochondrial membrane potential and cytochrome c release caused by ethanol, and inhibits hepatocyte apoptosis [67].

Heat stress is a nonspecific defense response of the body to high temperatures. When heat stress occurs, the expression of cytokines changes significantly, the immune system is dysregulated, inflammation occurs, and a large number of free radicals are produced, resulting in oxidative damage. L-theanine can play an antioxidant role by reducing MDA in liver tissue, increasing SOD, GSH-Px, and CAT enzyme activities, and improving tissue damage and oxidative stress caused by heat stress in mice. It also improves nutrient digestion and absorption, reduces the expression of inflammatory cytokines including TNF-α, IL-6, and IFN-γ, and alleviates the damage to the jejunum and liver tissue caused by heat treatment. The mechanism is that high temperatures and inflammatory factors can activate the P38-mitogen-activated protein kinase (MAPK) signaling pathway, and the expression levels of apoptosis signal-regulating kinase 1 (ASK1) and MAPK-activated protein kinase 2 (MK2) proteins are significantly up-regulated, which can be inhibited by L-theanine. The expression levels of MAP kinase kinase (MKK) 3 and MKK6 proteins were significantly down-regulated, and L-theanine could significantly activate MKK3 and MKK6 proteins to normal levels. L-theanine also alleviates the downregulation of p-P38/P38 phosphorylation and the decrease of MSK1 activity caused by heat stress, thereby regulating the P38-MAPK signaling pathway. Under heat stress and inflammatory conditions, the body preferentially synthesizes the heat shock protein HSP27, and the NF-κB signaling pathway is activated. L-theanine supplementation could decrease the overexpression of HSP27 and p-p65/p65 and exert anti-inflammatory, anti-oxidative, and immunomodulatory effects [68].

For DOX-induced oxidative damage and related adverse reactions, L-theanine plays an antioxidant role and reduces the side effects of chemotherapy by increasing the glutamate content and restoring the GSH level in the heart and liver [69]. Excessive dopamine induces neurotoxicity, while L-theanine could increase GSH levels, weaken the formation of quinolone proteins in midbrain neurons, and protect against oxidative stress and neuronal damage [11].

### 4.3. Regulation of Cytokines and Neurotransmitters

Cytokines are a class of small molecular proteins produced by immune cells that can transmit information between cells and have immunomodulatory and effector functions. Previous studies have shown that L-theanine has a regulatory role in immune cytokines, neurotransmitters, and hormone secretion. Li et al. [18] administered L-theanine to rats by gavage and found that the content of IL-4, 6, and 10 and the ratio of IL-4 to IFN-γ in serum decreased. This indicates that L-theanine can adjust the Th2/Th1 cytokine balance to shift toward Th1 and enhance resistance to pathogens. Hormones secreted by the neuroendocrine system can regulate immune function, and immune responses can also regulate the neuroendocrine system. After being absorbed by the human body, L-theanine can penetrate the blood-brain barrier and regulate the secretion of neurotransmitters in central nervous system cells. L-theanine administration increases the levels of dopamine and serotonin in the pituitary and hippocampus, leading to a decrease in serum cortisol levels, which enhances immune function.

Excitotoxicity in the central nervous system is mediated by excitatory neurotransmitters, and glutamate is the most important excitatory neurotransmitter in the central nervous system. Since L-theanine is the analog of glutamate, L-theanine may compete with glutamate for glutamate receptors, thereby inhibiting the excitotoxicity of glutamate and providing neuroprotection. L-glutamate triggered Abeta neurotoxicity in human APP (Swedish mutation) transgenic SH-SY5Y cells, and L-theanine could attenuate L-glutamate-induced apoptosis and activation of c-Jun N-terminal kinase and caspase-3. These results suggested that L-theanine had the potential for the prevention and treatment of Alzheimer’s disease [70].

L-theanine treatment did not affect the proliferation and division index of splenic lymphocyte subsets in rats. However, L-theanine could regulate the levels of IFN-γ, IL-2, IL-4, IL-10, IL-12, and TNF-α, and increase the mRNA and protein expression of RAS-related proteins, including Rap1A, HMGCR, and FDPs. In addition, Rap1A and HMGCR protein expression was positively correlated with IFN-γ, IL-4, and IL-6 levels. These results suggested that L-theanine may regulate cytokine secretion in rat splenic lymphocytes by activating the expression of Rap1A and HMGCR proteins involved in the mevalonate biosynthesis pathway [62].

In a mouse model of lipopolysaccharide (LPS)-induced acute liver injury, L-theanine improved the LPS-induced systemic inflammatory response, inhibited the production of IL-1β, TNF-α, and IL-6, and improved the LPS-induced immune imbalance. The specific mechanism is that L-theanine downregulates adrenocorticotropic hormone (ACTH) and corticosterone (CORT0), normalizes the excessive activity of the hypothalamic-pituitary-adrenal (HPA) axis, weakens the phosphorylation of NF-κB in liver tissue, controls the inactivation of the NF-κB signaling pathway, and thus inhibits an inflammatory state [71].

L-theanine regulates glutamine metabolism and immune function by competitively binding to cannabinoid receptor 1. In normal mice, L-theanine inhibited the phosphorylation of extracellular regulated kinase (ERK) 1/2 by antagonizing cannabinoid receptor 1, thereby affecting glutamine synthetase (GS) expression. From the perspective of immune signaling, L-theanine antagonized the activity of cannabinoid receptor 1, relieved the inhibition of cannabinoid receptor 1 on COX-2 expression, down-regulated Pdcd4 expression and NFκB, and finally enhanced the expression of the anti-inflammatory factor IL-10. In E44813-stressed rats, L-theanine promoted the nuclear translocation of p-ERK1/2 by inhibiting cannabinoid receptor 1 activity, which ultimately acted on GS (a key enzyme in glutamine metabolism). Meanwhile, it can reduce the expression of the pro-inflammatory factor TNF-α and increase the expression of the anti-inflammatory factor IL-10 in stressed rats through COX2-Pdcd4-NFκB-IL10 and TNFα pathways [72].

## 5. Application of L-Theanine as an Immunomodulator in the Animal Industry

### 5.1. Immunomodulation in Poultry

L-theanine improves the anti-stress ability and immunity of animals and could be used as a green feed additive in animal production. Supplementation of L-theanine in the diet improves muscle antioxidant status and meat quality in broiler chickens [73,74]. L-theanine can alleviate the immune stress response caused by LPS, maintain the α1-acid glycoprotein (α1-AGP) and IL-6 concentrations in serum, and the secretory immunoglobulin A (sIgA) content in the jejunal mucosa [75], inhibit the infection and survival of coccidia in vivo, and improve the non-specific immune response of chickens [76].

Dietary supplementation of L-theanine decreases serum total cholesterol content and increases HDL in broilers. It also reduces the content of IL-2 and INF-γ in serum, the mRNA expression of TNF-α and IL-6 in the thymus, and the mRNA expression of IFN-γ and IL-2 in the spleen. In addition, supplementation with 200 mg/kg L-theanine improves the antioxidant status in the blood by increasing the levels of SOD, GSH-Px, and CAT, thus enhancing the antioxidant capacity and reducing acute oxidative stress in broilers [77]. Further studies have shown that L-theanine significantly increases the beneficial microorganisms in the ileum and jejunum, reduces harmful microorganisms, increases the height of the jejunal villus, enhances the content of tight junction protein in the duodenum, and improves the intestinal mucosal barrier. These data showed a positive effect of L-theanine on intestinal immunity in broilers [78].

It has been proven that L-theanine is safe for the later growth of yellow-feathered broilers. It increases the contents of intestinal sIgA and serum IL-2 in the early stage and later stage of growth, respectively, and enhances the content of serum IFN during the whole growth period [79]. The same experimental results were also observed in meat ducks. L-theanine could improve the growth performance and relative weight of immune organs in meat ducks by enhancing antioxidant capacity. L-theanine increased the contents of IgM, IgG, and IgA and decreased the levels of IFN, TNF-α, IL-2, and IL-6. L-theanine also improved jejunal morphology and increased villus height, V/C, and goblet cell number. All these results indicated that L-theanine is a potential feed additive for improving poultry immunity [80].

### 5.2. Immunomodulation in Livestock

Skeletal muscle is a complex, heterogeneous tissue composed of muscle fibers. According to the myosin heavy chain (MyHC) subtype, muscle fibers can be divided into oxidative slow muscle fibers (type I) and glycolytic fast muscle fibers (types IIa, IIx, and IIb). Dietary L-theanine supplementation promotes the transformation of skeletal muscle fibers from type II to type I and improves pork quality in weanling piglets, which may be through improving antioxidant capacity and mitochondrial biogenesis and activating the calcineurin signaling pathway [81]. After stimulation by LPS, the growth performance, antioxidant capacity, and immune function of weaned piglets significantly decreased. L-theanine supplementation significantly increased the daily gain and feed intake of weaned piglets, enhanced the contents of GLU, IgA, IgM, and IgG, and the activity of GSH-Px, and decreased the contents of COR, MDA, and IL-10 in serum. These suggested that L-theanine could protect against oxidative damage and improve the growth performance and immune function of weaned piglets [82]. Similarly, L-theanine and beta-glucan alone or in combination could inhibit the production of pro-inflammatory cytokines, increase the levels of anti-inflammatory cytokines, reduce the inflammatory response against bacterial infection, and enhance immune function in weanling piglets stimulated with LPS [83].

L-theanine decreases the level of D-lactate and TNF-α in serum and inhibits the phosphorylation of ERK1/2, MAPK, and JNK in piglets [84]. In addition, L-theanine pretreatment can alleviate diquat-induced oxidative stress of the intestinal barrier and increase the expression of tight junction proteins such as zonula occludens 1, claudin 1, and occludin in the jejunum and ileum of piglets. Moreover, it can inhibit the expression of TNF-α, IL-1β, and IL-6, reduce the intestinal mucosal inflammatory response, and improve the intestinal barrier function of weanling piglets by inhibiting the TLR4/p38 MAPK/NF-κB signaling pathway [85].

Heat stress causes an increased LPS level and related inflammatory responses in lactating cows. L-theanine can reduce the rectal temperature of dairy cows, affect lipid metabolism, reduce the translocation of LPS to peripheral blood and the accumulation of LPS in milk during heat stress, and decrease the content of IL-1β and the inflammatory response induced by LPS in dairy cows [10,86].

## 6. Concluding Remarks

**Figure 1:**
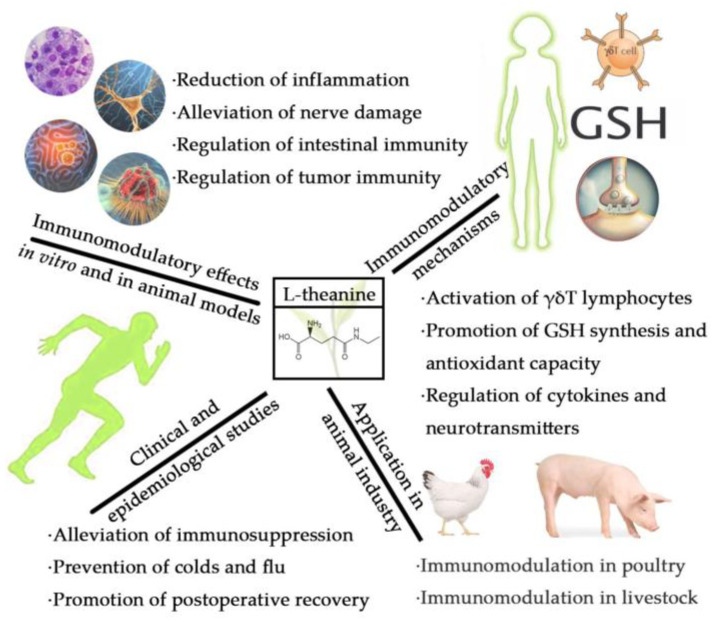
Immunomodulatory effects of L-theanine and the related mechanisms in vitro and in vivo.

L-theanine is an effective and safe natural compound with immunoregulatory activity. Clinical and epidemiological research has proved that L-theanine could alleviate immunosuppression caused by strenuous exercise and prevent colds and flu by strengthening immunity. Extensive cellular and animal studies have shown that theanine regulates immune function in a variety of conditions, such as inflammation, nerve damage, intestinal tract disease, and tumors. Core mechanisms for improving immunity include the improvement of T lymphocyte function, promotion of GSH synthesis and antioxidant capacity, and regulation of cytokines and neurotransmitters. Dietary supplementation of L-theanine as a safe immune enhancer has good application prospects in poultry and animal husbandry. The immunomodulatory effects of L-theanine and the related mechanisms in vitro and in vivo are summarized in Figure 1. Future investigation should focus on further enhancing the immune activity of theanine through chemical modification, embedding, delivery, and combination with other compounds. This is of great significance for expanding the application of theanine as an immunomodulator in humans and animals.

## Figures and Tables

**Table 1:** Major physicochemical properties of L-theanine.

Physicochemical Property	Description
Molecular formula	C_7_H_14_N_2_O_3_
Molecular weight	174.198 g/mol
Melting point	207 °C
Density	1.2 ± 0.1 g/cm^3^
Appearance	Crystalline solid
Solubility	Soluble in water and insoluble in ether, alcohol
Taste	Odorless, umami, and sweet taste
Stability	Stable in acidic and unstable in alkaline conditions

## Data Availability

Not applicable.
